# Hidrocystoma of the external auditory canal: a case report

**DOI:** 10.1186/1757-1626-2-79

**Published:** 2009-01-22

**Authors:** Dimitris G Ioannidis, Emmanouil I Drivas, Chariton E Papadakis, Antzela Feritsian, John G Bizakis, Charalampos E Skoulakis

**Affiliations:** 1 Department of Otorhinolaryngology, General Hospital of Volos, Magnesia, 38222, Greece; 2 Department of Otorhinolaryngology, University of Crete School of Medicine, Heraklion, Crete, Greece; 3 Department of Histopathology, General Hospital of Volos, Magnesia, 38222, Greece

## Abstract

**Background:**

Apocrine hydrocystomas arising in the external auditory canal are very rare. In this report a clinical case of apocrine hydrocystoma located in the cartilaginous part of the external auditory canal is presented.

**Case presentation:**

A 64-year-old Caucasian female patient presented with a solitary nodule, located in the outer part of the external ear canal after repeated episodes of external otitis. For diagnostic purposes, computerized tomography was used. The patient underwent an excisional biopsy of the mass via an intra-aural incision and the surgical specimen was sent for histopathologic examination.

**Conclusion:**

An apocrine hidrocystoma inside the auricular canal is uncommon. It can cause recurrent external otitis and conductive hearing loss and should be treated with wide local excision and reconstruction of the external auditory canal for diagnostic and therapeutic purposes.

## Background

The lateral one-third of the external auditory canal is cartilaginous and covered with a thin epithelium, firmly bound to the perichondrium and with scant subcutaneous tissue [1]. Hair follicles formed by invagination of epidermis are numerous in the outer one-third of the cartilaginous canal but are less noumerous in the inner two-thirds. Sebaceous glands are plentiful and open into the follicles of fine vellus hair. Eccrine sweat glands are not present in the auditory canal, but it is abundantly supplied with modified (ceruminous) glands. The number of these glands varies considerably, in individuals and in many races, with the primary concentration in the cartilaginous part of the canal, but with some sparse distribution in the osseous portion. These glands secrete at a very low, constant rate. Histologicaly, the apocrine glands lie deep in the dermis adjacent to the cartilage. They are comprised of an inner columnar, eosinophilic epithelium which is surrounded by an outer layer of myoepithelial cells. The secretion of the glands is strongly PAS positive and is at times stored in the gland lumina in sufficient quantities to cause glandular dilatation. Whereas the function of eccrine sweat glands is to help regulate the body heat and fluid, the apocrine sweat glands have this role to a lesser degree and indeed, in the ear canal, have a more protective function. Cerumen itself derives from the sebaceous and apocrine glands of the ear canal. The acidity of these secretion keeps the pH of the auditory canal between 5,6 – 5,8 near the concha and to 7,3 – 7,5 at a distance of 5–10 mm within the canal. This acidity helps combat many organisms implicated in external canal infections, most of which grow best in an alkaline medium with a pH between 7,2 – 7,6. In addition, these glandular secretions provide a protective water proof coating for the skin. These functions, along with the natural lateral migration of the meatal skin at the rate of 1,5 mm per month, play an important part in the antimicrobial defense mechanism of the ear canal [1].

Hidrocystomas are rare, benign skin adnexal neoplasms, sometimes presenting as cystic lesions [2]. According to the histologic characteristics and presumed histogenic derivation, hidrocystomas have been categorized into two types (apocrine and eccrine). Eccrine hidrocystomas present as small, tense, thin-walled cysts, ranging from 1–6 mm in diameter, and can occur as single or multiple lesions. They are found predominantly in adult females, are located mostly on the periorbital and malar regions, and are prevalent in adults between 30 and 70 years of age. Apocrine hidrocystomas arise from the proliferation of apocrine glands and are usually solitary, with a diameter of 3–15 mm. Apocrine lesions are also found mostly on the head and neck and along the eyelid margin near the inner canthus. The general distribution of lesions tends to occur in similar locations on the body for both types of hidrocystomas [[2,3], and [4]].

We describe a patient with an apocrine hidrocystoma in the auricular canal. In the English literature, very few cases of apocrine hidrocystomas originating in the external auditory canal have been reported. A single, related case has been identified in the PUBMED database [3] of an apocrine hidrocystoma involving the pinna and another one is reported in the Iranian Red Crescent Medical Journal [5]. The clinical and histological features, the differential diagnosis, and the treatment of apocrine hydrocystoma are discussed.

## Case presentation

**Figure 1:**
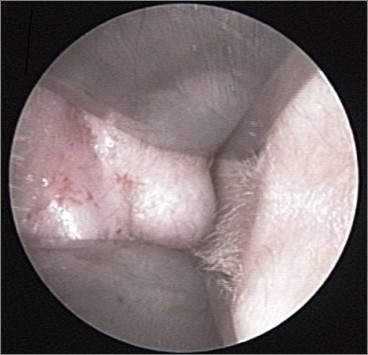
**Photographic image of the apocrine hidrocystoma inside the auricular canal with the use of a rigid 0° 6-cm endoscope**.

**Figure 2:**
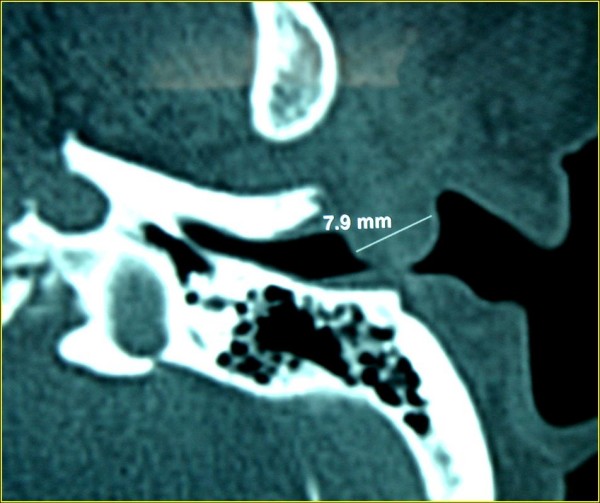
**Axial, soft tissue window CT scan demonstrating a polypoid soft tissue mass located at the posterior canal wall extending from the entrance to the bone-cartilage limit of the auditory canal, 7.9 mm in diameter**.

A 64-year-old Caucasian female presented to our department with slowly developing left side hearing loss and recurrent external otitis. Physical examination revealed a tender solitary, skin-colored, dome-shaped nodule arising from the inferior and posterior wall of the left external auditory canal that narrowed the external auditory meatus and canal (figure 1). Pure tone audiometry revealed a mild conductive hearing loss on the left side. Computerized tomography (CT) scan of the temporal bone (figure 2) demonstrated a well-circumscribed, soft tissue narrowing most of the external auditory canal. The mass arised from the inferior and posterior canal wall at the cartilaginous portion of the external auditory canal, with no bone erosion and no middle ear or mastoid involment.

The patient underwent, under local anesthesia, an excisional biopsy of the mass via an intra-aural incision. The mass was removed while preserving the osteocartilaginous canal wall and the integrity of the underlying tympanic membrane. The external auditory canal was packed with a Merocel wick to support the skin and to prevent canal stenosis for two weeks. The patient made an uneventful recovery and there was no recurrence during two years of follow-up.

**Figure 3:**
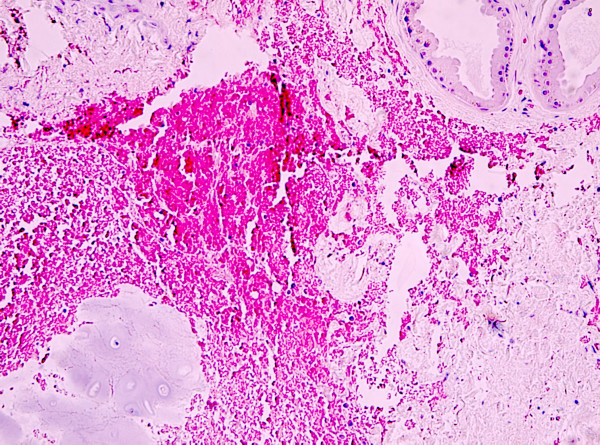
**Histopathologic section of the surgical specimen demonstrating a cyst in the dermis lined by a one-or two-layered cuboidal or flattened epithelium resting on a layer with spindle myoepithelial cells**. In close vicinity to the cyst are groups of apocrine glands, partly dilated, showing the typical eosinophilic epithelium with evidence of decapitation secretion (H&E, original magnification 10×).

Histopathologic examination of paraffin sections of the specimen, stained with haematoxylin-eosin (figure 3), revealed the presence of a cyst in the dermis lined by a one-or two-layered cuboidal or flattened epithelium resting on a layer with spindle myoepithelial cells. In close vicinity to the cyst were groups of apocrine glands, partly dilated, showing the typical eosinophilic epithelium with evidence of decapitation secretion, the pathognomonic finding of apocrine differentiation [[6,7], and [8]]. The deepest group of glands was situated close to mature cartilage. Immunohistochemistry revealed epithelial membrane antigen (EMA) expression in all the cells of the cyst wall, while carcinoembryonic antigen (CEA) focally-decorated the luminal cells. Smooth muscle actin (SMA) was present in the basal layer. Human milk fat globulin 1 (HMFG) and gross cystic disease fluid protein 15 (GCDFP-15), which is regarded as a marker of apocrine epithelium [7,8], were weakly positive in some cells of the cyst wall, showing decapitation secretion, while being strongly positive in the cells of all of the above described glandular groups. These light microscope findings were considered diagnostic of the apocrine nature of the lesion in question.

## Discussion

Skin adnexal neoplasms exhibit morphological differentiation towards one or more types of adnexal structures found in normal skin (pilosebaceous units, eccrine glands, and apocrine glands) and comprise a wide spectrum of benign and malignant tumours, including cylindromas, spiradenomas, trichoepitheliomas, trichoblastomas, sebaceous adenomas, sebaceomas, sebaceous carcinomas, syringomas, microcystic adnexal carcinoma, papillary eccrine adenomas, tubular apocrine adenomas, hidradenomas, poromas, digital papillary adenomas/adenocarcinomas, adenoid cystic carcinomas, syringoid/eccrine carcinomas, apocrine (axillary) carcinomas, eccrine and apocrine hidrocystomas, and admixtures of these entities [6]. Most adnexal neoplasms are uncommonly encountered in routine practice, and pathologists can recognise a limited number of frequently encountered tumours.

McCalmont [6] offered a logical and systematic approach to the classification of adnexal neoplasms by dividing these lesions into two main diagnostic categories: 1) eccrine and 2) folliculosebaceous-apocrine. The cornerstone of this classification scheme derives from the embryology of epidermal development.

Most adnexal tumors are benign, but many have definite recurrence and malignant potential if incompletely excised. Therefore, the presence of any atypical or malignant features, such as asymmetric growth, jagged/infiltrative borders, irregular arrangement of neoplastic cells, cytonuclear atypia, increased mitotic activity, tumour necrosis, and superficial ulcerations in a given case should be accessed, and complete excision for lesions that are only partially sampled is recommended to facilitate complete evaluation and to prevent future recurrence and rare malignant transformation [6,7].

Both benign and malignant tumors grow along the path of least resistance, extending laterally out the meatus or medially through the tympanic membrane and into the middle ear [9]. Invasive malignant tumors also extend radially through auricular cartilage and the fissures of Santorini into the parotid gland or into surrounding periauricular tissue. Medial extension with erosion of tympanic membrane or temporal bone is also indicative of malignancy. Diagnosis is made on the basis of microscopic examination of biopsy specimens. To distinguish between benign and malignant tumors, biopsy specimens should be large enough to identify deep tissue invasion. HRCT of the temporal bone and MRI of soft tissue around the lateral skull base can be helpful in this respect also [9,10]. Benign tumors are treated with wide local excision and reconstruction of the external auditory canal. Because of high recurrence rates, especially in cases of adenoid cystic carcinoma, treatment of malignant lesions should be aggressive. Lateral temporal bone resection with parotidectomy and postoperative radiation therapy is appropriate for early lesions. Larger malignant tumors require a more extensive temporal bone resection and cervical lymph node dissection in addition to parotidectomy and postoperative radiation therapy [9,10].

## Conclusion

We have described an unusual appearance of an apocrine hidrocystoma inside the auditory canal of a female patient that caused occlusion, conductive hearing loss and secondary otitis externa. Although diagnosis relies on biopsy with histologic examination, diagnostic imaging by CT-scan is necessary for defining the characteristics, extension of the lesion and for planning the therapeutic approach. Localized excisional biopsy is the treatment of choice for external auditory canal apocrine hydrocystomas via intra aural or rertoauricular approach while preserving the adjacent structures.

## Abbreviations

ENT: ear, nose, throat; CT: computerized tomography; FNA: fine needle aspiration; PAS: periodic acid Schiff; CEA: carcinoembryonic antigen; EMA: epithelial membrane antigen; GCDFP-15: gross cystic disease fluid protein 15; HMWK: high molecular weight keratin; LMWK: low-molecular weight cytokeratin; SMA: smooth muscle actin

## Consent

Written informed consent was obtained from the patient for publication of this case report and accompanying images. A copy of the written consent is available for review by the Editor-in-Chief of this journal.

## Competing interests

The authors declare that they have no competing interests.

## Authors' contributions

CES was responsible for diagnosing and performing surgery on the patient. DGI drafted and prepared the manuscript. CEP was responsible for review of the literature. EID reviewed the patient's medical record in order to collect all the available information. JGB was involved in revising the article for intellectual details. AF performed the histologic examination of the surgical specimen. All authors read and approved the final manuscript.
